# Inferior STEMI Electrocardiogram in a Young Postpartum Female with Sickle Cell Trait with Chest Pain - A Case Report

**DOI:** 10.21980/J8KP95

**Published:** 2022-10-15

**Authors:** Jessica Truong, Ryan Perdomo, Daniel Ng, Sassan Ghassemzadeh, John Costumbrado

**Affiliations:** * University of California, Riverside School of Medicine, Riverside, CA; ^ Riverside Community Hospital, Department of Emergency Medicine, Riverside, CA

## Abstract

This is a case of a 35-year-old postpartum female presenting to the emergency department (ED) with acute onset chest pain and dyspnea with initial concern for acute ST-segment elevation myocardial infarct (STEMI). Her electrocardiogram (ECG) showed ST segment elevations in the lateral leads and inferior leads with depressions in the high lateral and septal leads. Given her post-partum status, spontaneous coronary artery dissection (SCAD) was part of the differential as well as other emergent processes. Upon cardiac catheterization, the patient was found to have 50% vasospasm in the left circumflex coronary artery. Her post-catheterization echocardiogram showed a normal ejection fraction (EF) without regional wall abnormalities. This is an interesting case of chest pain in a younger patient, without typical acute coronary syndrome (ACS) risk factors, presenting with a concerning ECG likely due to coronary vasospasm. We will discuss other atypical causes of STEMI patterns that should be considered in the differential and management of such patients.

**Topics:**

Sickle cell trait, STEMI, postpartum, vasospasm, ECG, cardiology.

**Figure:**
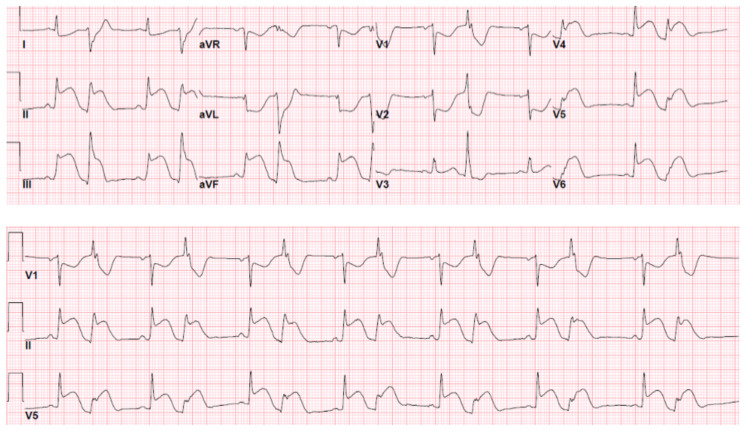


## Brief introduction

Acute coronary syndrome is an emergent manifestation of chest pain that includes STEMI, non-ST-segment elevation myocardial infarction (NSTEMI), and unstable angina.^1^ Common risk factors include diabetes, hypertension, hyperlipidemia, obesity, and smoking.^1^ Family history of cardiovascular disease is also an important consideration.^1^ While often thought of as a process that occurs in patients over 50 years, younger patients can also be impacted.^2^ In younger patients without the typical ACS risk factors, processes such as coronary vasospasm must be considered in the differential,^2^ in addition to rarer causes such as SCAD.^3^

This is an interesting case of chest pain in a younger patient with a STEMI pattern given her lack of typical ACS risk factors. She presented with risk factors that may be contributory to her presentation, including a history of sickle cell trait and recent pregnancy. In the case presented, although her clinical presentation and ECG findings were a result of coronary vasospasms, SCAD is a diagnosis that should be considered in any young patient, particularly women, presenting with a clinical picture of STEMI without a history of coronary artery disease or its risk factors.

## Presenting concerns and clinical findings

We describe a 35-year-old, G11P6A3M2, female who presented to the emergency department with severe chest pain of acute onset with associated dyspnea. Patient’s medical history was remarkable for sickle cell trait and recent uncomplicated vaginal delivery two weeks prior to the emergency department visit. Patient’s vitals and physical exam were unremarkable. ECG showed evidence of ST segment elevation concerning for acute STEMI. Her chest x-ray was interpreted as normal with no acute intrathoracic abnormality. Her initial troponin was 0.345 ng/mL (0.015 – 0.045 ng/mL). Interventional cardiology was consulted, and the patient emergently underwent cardiac angiography with the differential including but not limited to thrombosis, SCAD, and coronary vasospasm. Findings of coronary vasospasm on cardiac catheterization include total or subtotal obstruction or severe diffuse vasoconstriction of epicardial coronary artery associated with transient myocardial ischemia (MI) as evidenced by ischemic ST-segment changes on ECG.^4^ Severe diffuse vasoconstriction per the American Heart Association classification is defined as 90% stenosis seen in >2 adjacent coronary segments of epicardial coronary arteries.^4^ During cardiac catheterization, interventional cardiology noted improved vasospasm and increased flow after treating with intracoronary nitroglycerin.

## Significant findings

ECG shows evidence of ST segment elevation in the inferolateral leads with reciprocal change in a bigeminy pattern. The ECG pattern seen in this patient demonstrates ST elevations in the inferior leads (II, III, and avF) as well as the precordial leads V4–V6. Reciprocal changes can also be seen in leads I and avL. Though this STEMI pattern is typically associated with occlusion of the right coronary artery in 80% of cases, it may also be caused by occlusion of the left circumflex artery.^1^ This may explain this patient’s cardiac catheterization findings of vasospasm in the left circumflex coronary artery.

## Patient course

Upon cardiac angiography, the patient was found to have 50% vasospasm in the left circumflex coronary artery and no significant atherosclerotic disease or stenosis. Although there was concern for SCAD, there was no evidence of dissection. The patient was given intracoronary nitroglycerin with some resolution of the spasm and then started on aspirin and a heparin drip in the hospital. She later underwent an echocardiogram that showed an ejection fraction of 60–65% without regional wall abnormalities. The patient improved throughout her hospital course and was discharged in stable condition two days later with amlodipine (for vasospasm), aspirin, atorvastatin, metoprolol, and isosorbide mononitrate with recommendations to follow up in cardiology clinic in one week.

## Discussion

Despite SCAD existing as an initial concern during catheterization given the patient’s risk factors, the presentation was found to be more consistent with coronary artery vasospasm. Vasospastic angina (previously referred to as Prinzmetal or variant angina) is defined by episodes of angina at rest that occur because of coronary artery vasospasm with characteristic ST-segment elevations seen on ECG.^5^ Coronary artery vasospasm causes a decrease in the blood supply to the myocardium, followed by resultant ischemia and anginal symptoms, with infarction occurring if this spasm persists.^6^,^7^,^8^ Vascular smooth muscle hyper-reactivity within the coronary arteries is thought to be central to the cause of vasospastic angina.^5^,^6^,^7^ Vasospasm triggers include cold weather, exercise, exposure to alpha-agonists that promote vasoconstriction (eg, pseudoephredrine), recreational drug use (eg, cocaine, amphetamines), alcohol use, and physical/emotional stress.^8^,^9^ Patients with vasospastic angina tend to present with episodic chest pain lasting 5–15 minutes.^8^ Vasospastic angina typically occurs from the hours of midnight until the early morning due to increases in both sympathetic and parasympathetic nervous system activity; nocturnal increases in acetylcholine and adrenergic activity and decreases in vagal tone have been noted to contribute to the time frame observed.^10^ This type of chest pain is not exertional in nature or alleviated with rest, and it is relieved with use of short-acting nitrates.^8^ Ischemic ST-segment changes on ECG may be seen during an episode but resolve when symptoms resolve.^8^ Treatment includes pharmacologic therapy that targets the coronary vasculature, such as calcium channel blockers and long-acting nitrates.^8^

Risk factors for vasospastic angina include smoking and insulin resistance.^9^ Additionally, a systematic review of cases of acute myocardial infarction from coronary artery vasospasm in pregnancy or the postpartum period showed that patients experiencing this condition tend to be of advanced maternal age, multigravida, and in their third trimester or postpartum.^11^ This review suggests that the postpartum period may be a risk factor for coronary artery vasospasm, which may explain our patient’s presentation, ECG interpretation, and cardiac catheterization findings. Although the patient in this case is not within the typical age range for diagnosis of vasospastic angina, her female sex and recent postpartum status upon presentation to the emergency department, the latter of which is often associated as a physically and emotionally stressful time for mothers^12^, may have increased her risk for vasospastic angina.

It is worth considering that the patient’s history of sickle cell trait may also be an independent risk factor for the STEMI pattern seen, because it predisposes her to experiencing vaso-occlusive crises under the appropriate conditions.^13^ Sickle cell trait does not typically cause the characteristic vaso-occlusive crises seen in sickle cell disease, unless patients are exposed to conditions that promote sickling, such as severe hypoxia, dehydration, increased sympathetic outflow, hypothermia/hyperthermia, high 2,3-DPG levels, and the release of inflammatory cells.^13^ The characteristic sickle shape makes red blood cells less elastic and mobile as they travel through capillaries.^13^ Sickle cells accumulate in areas of vessel narrowing, which results in activation of inflammatory responses that promote endothelial damage.^13^ The combination of sickle cell accumulation and endothelial dysfunction contributes to the vaso-occlusion associated with sickle cell disease and trait.^13^ The absence of coronary artery atherosclerotic disease seen in autopsy reports on patients with sickle cell disease suggests vaso-occlusion as the likely cause of myocardial ischemia and injury in these patients.^14^ Management of MI in sickle cell disease targets vaso-occlusive injury, including red cell transfusions and nitric oxide.^14^ Although this mechanism of myocardial ischemia is associated with sickle cell trait/disease, the current literature does not support an association between sickle cell trait and increased risk of MI, at least in African American individuals.^15^

Though an infrequent cause of MI that accounts for less than 1% of all acute MIs,^16^ spontaneous coronary atery dissection (SCAD) was still important to consider in the differential diagnosis for the patient discussed in this case. Spontaneous coronary artery dissection (SCAD) is caused by a non-traumatic and non-iatrogenic separation of the epicardial coronary artery wall due to intramural hemorrhage.^16^ Myocardial infarctions secondary to SCAD can be treated with interventions including percutaneous coronary intervention and coronary artery bypass grafting; medical management typically consists of anticoagulation, dual-antiplatelet therapy, and beta blockers.^17^ Chest pain is the most common presenting symptom in 85–96% of cases with variable radiation to the arm, neck, or back.^16^ Potential triggers of SCAD include emotional stress, physical stress, use of stimulant medications or recreational drugs, and hormonal triggers, such as pregnancy.^16^ Although 90% of patients with SCAD are women between the ages of 47–53,^16^ postpartum status has been reported in 2–18% of cases of SCAD.^3^,^18^,^19^ Peripartum women are thought to be at increased risk for SCAD due to increased physiologic hemodynamic stressors in addition to hormonally mediated weakening of coronary arterial walls.^20^ Furthermore, exposure to recurrent and chronic hormonal pregnancy changes can increase the risk of SCAD in multiparous women.^21^ Although the patient in this case is outside the typical age range associated with SCAD, her postpartum status may have been a risk factor for this rare cause of MI.

The diagnosis of SCAD is made in most patients with the use of coronary angiography.^18^ The angiographic definition of SCAD includes the presence of a non-iatrogenic dissection plane in the absence of coronary atherosclerosis with typical changes of radiolucent intimal flap and contrast staining; these findings, however, are seen in less than 30% of non-atherosclerotic SCAD cases.^18^,^19^ The patient in this case was 2 weeks postpartum and had a cardiac catheterization revealing approximately 50% left circumflex distal spasm with no atherosclerotic or stenotic disease. Given these findings, SCAD could not be completely excluded from the differential in the causes of her chest pain; however, there is more evidence for coronary vasospasm at this time.

Vasospastic angina is more prevalent in females than males, although the overall incidence and prevalence is not currently known, and most patients are diagnosed between 40 and 70 years of age.^8^,^9^ Vasospastic angina accounts for 2% of cases of angina.^23^ Meanwhile, overall prevalence of myocardial infarction in females aged 35 to 44 is 5.2%, and annually, more than 30,000 women younger than 55 are hospitalized for MI.^22^ In comparing men and women, women below the age of 55 tend to have greater hospital lengths of stay and in-hospital mortality.^22^ Despite how rare vasospastic angina and SCAD are in young female populations, both are still important diagnoses to consider in the differential for a young woman presenting with anginal chest pain, particularly those with risk factors including postpartum status and medical history such as sickle cell trait. This case highlights the importance of causes of ACS such as SCAD, which was an initial concern during this patient’s diagnostic and therapeutic cardiac catheterization. Follow up information or clinical course is unavailable at the time of the submission of this case report.

## Supplementary Information




