# Supplementary figures and images for: Inferior STEMI Electrocardiogram in a Young Postpartum Female with Sickle Cell Trait with Chest Pain - A Case Report
Source: J Educ Teach Emerg Med. 2022 Oct 15;7(4):V10–4. doi: 10.21980/J8KP95 (PMC10332670; doi:10.21980/J8KP95)

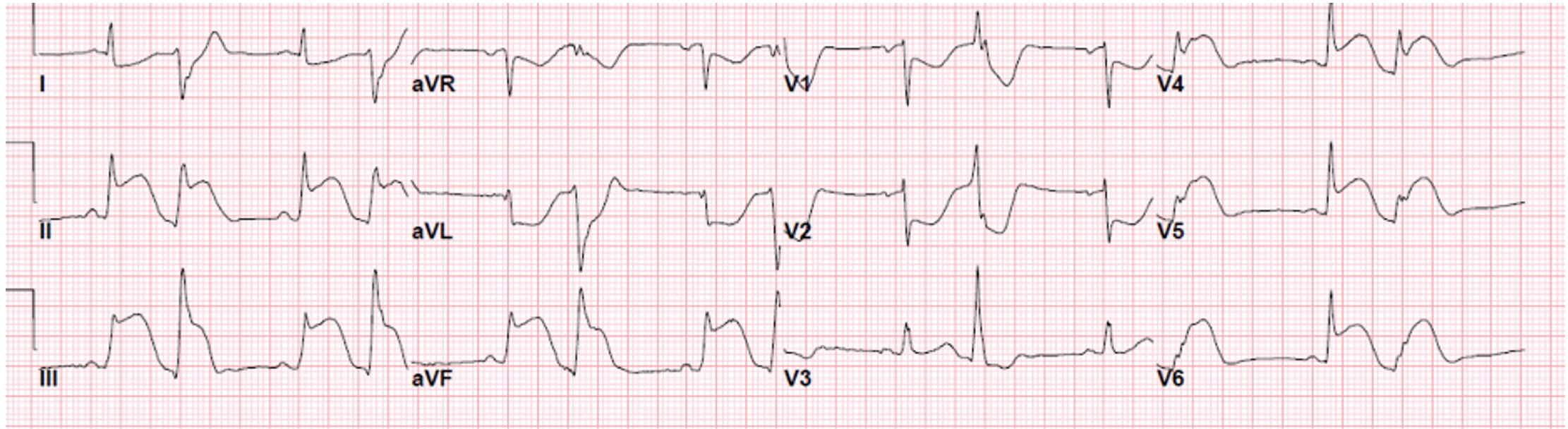

Supplement: Supplementary file 1 [file JETem-7-4-V10-supp1.jpg]

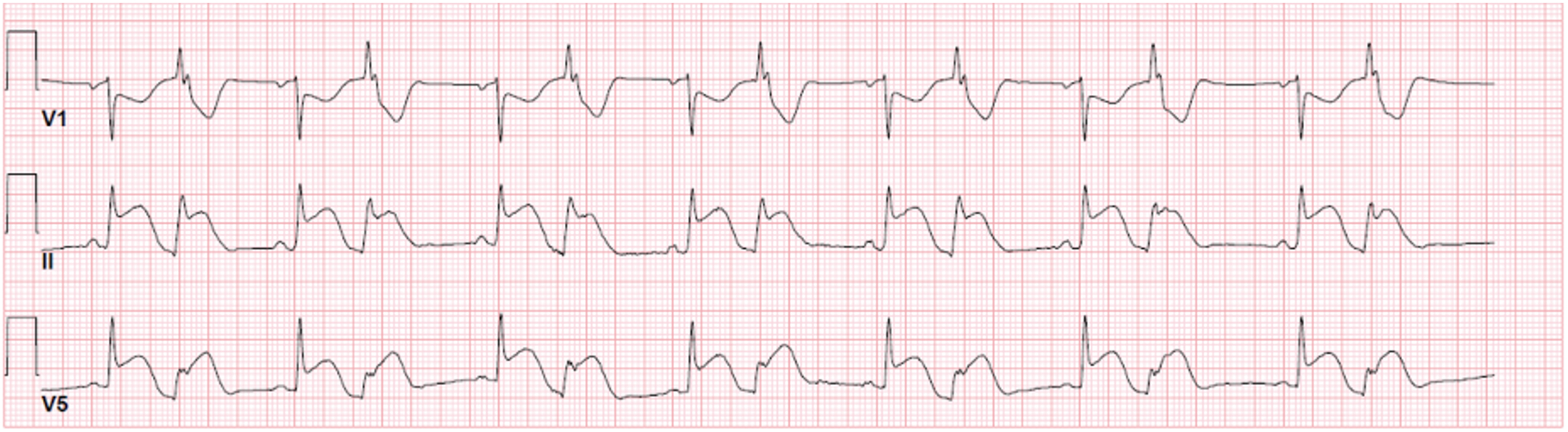

Supplement: Supplementary file 2 [file JETem-7-4-V10-supp2.jpg]
